# Introductory

**Published:** 1844-04

**Authors:** 


### ILLINOIS
### MEDICAL & SURGICAL JOURNAL.

VOL. I. APRIL, 1844. NO. 1.

### INTRODUCTORY.

It is with pleasure, if not with pride, that we announce to the medical public, the first number of the first Medical Journal that has been issued in the State of Illinois. Such an announcement may perhaps, by some, be thought premature. So indeed did it appear to us, when our attention was first called to the subject. More mature reflection, however, convinced us that such an opinion was erroneous; and that it is so, must appear to all, who will consider the condition of the Medical Profession in the West—their distance from the sources of improvement, and from each other, and the necessity of disabusing public opinion of the impositions of empirics, for the spread of whose doctrines new countries afford peculiar facilities.

The rapid strides which the profession is making in the improved means of diagnosis and the application of new remedies; in more easy modes of remedying deformity, and the increasing simplicity of surgical operations, not to mention other branches; require that means should be devised of more widely circulating medical information. It may be said that the periodicals already in circulation are adequate to the purpose. We would here remark, that nothing is farther from our design, than to enter into competition with the many excellent and more voluminous publications of other places; but rather, by making honorable mention of them, to give them their proper appreciation. We expect, however, that our columns will be read in portions of the West almost inaccessible to others. Country practitioners, in newly settled regions, are compelled to extend their practice over so wide a field that but little leisure is left them for reading. To them we hope our Journal will be acceptable, by presenting to them, in a condensed form, the practical hints and new views, which are more diffusely set forth in larger periodicals. The smaller size of our Journal enables us to offer it to them at a cost, which may come more within their means, while the shorter distance of mail carriage will materially lessen the amount of postage.

Our Journal will have, we hope, a local interest. It will be one of our main objects, to present to our readers, all information, that our exertions can obtain, of the types of diseases, and the modifications of treatment required by endemics peculiar to the particular sections in which it may circulate. To aid us in this endeavor, we invite practitioners to forward to us the result of their observations, whenever they may suppose them possessed of practical value. In regard to Epidemics also, statistics are desirable. The prevailing type of disease, its mortality, the comparative success of different modes of treatment, the point of its commencement and the direction of its progress through the infected district, and when it may be practicable, the Post Mortem appearances, will be points of value to those who may afterwards have to contest with the disease, and will form a record valuable in the medical history of the country.

We anticipate an advantage from our publication, in affording to Western Physicians means of giving to the public the results of their surgical operations, and other observations,—means not so readily afforded them by other Journals. They may thus, through the medium of our columns, become known to the profession generally, and to each other, and separated by space, a bond of union will be formed pleasant in the association, and powerful to repress empiricism.

Again, a condensed view of new publications will be given, and practitioners will be enabled to judge which of them will be most desirable as additions to their libraries.

In the prairies and forests of the West, there are, doubtless, to be found remedies whose properties, if known, would be invaluable. Investigations should be made with the view of developing these resources. It will always be a pleasure to us to receive any information regarding indigenous remedies, and, if accompanied with specimens, we will be happy to aid, with our best endeavors any advance in the improvement of the Materia Medica.

We have noticed with regret, the partial success of newfangled impostures, in giving a wrong direction to public opinion, where the faculty have not had sufficient organization to expose the false views of pretenders. It will be one of our objects, from time to time, to discuss such points of their pretensions as may appear most specious, and expose their sophistry. The authority of the most eminent medical writers and stubborn facts; and not our own unsupported opinions; will form the basis of all such discussions. The profession will thus have before them, the representations of the supporters of the so called "new systems," and their refutation; and will be better able to disabuse the public, and drive from the field charlatans, whose ignorance is only equalled by their presumption.

Besides these strictly local interests, our Journal will, we confidently hope, possess some interest to those at a distance from us. From a want of a proper vehicle, no information as regards Medical Practice in this part of the Union, can have found its way to the East, and doubtless, erroneous impressions are entertained. Many physicians, of talent and energy, would, without doubt, be desirous of settling in the North West, could they be assured of a proper appreciation of their efforts. To all such we will strive to furnish the information they may desire, and render them somewhat acquainted with the prevailing endemics with which they will have to cope. They will thus be prepared for extensive usefulness, after a much shorter residence in the place of their choice.

Our Journal at present covers but 16 pages. Should the patronage of the Profession afford sufficient encouragement, and circumstances render it advisable, our limits will be increased. We have around us three large States—Indiana, Michigan, and Illinois,—and two extensive Territories—Wisconsin and Iowa,—filled with medical men, of the highest intelligence, and most praiseworthy enterprise, and not a single Medical Journal has been previously issued in all this vast North Western region. Yet in this same region, political journals may almost be said to be without number; literary periodicals are numerous, and agricultural Journals perhaps equally so. They all meet with liberal patronage. It were strange indeed, if *one* Medical Journal should, if properly conducted, fail of success. Let the Physicians of the North West, urged by a common interest for the honor and advancement of the profession in their own region, aid us by their support, and we may hope that the day is not far distant, when the condition of medical science, will attain an equality, and keep pace with that in the other and older districts of the Union.

